# Error Rate Comparison during Polymerase Chain Reaction by DNA Polymerase

**DOI:** 10.1155/2014/287430

**Published:** 2014-08-17

**Authors:** Peter McInerney, Paul Adams, Masood Z. Hadi

**Affiliations:** ^1^Joint BioEnergy Institute, Emeryville, CA, USA; ^2^Sandia National Laboratories, Livermore, CA, USA; ^3^Physical Biosciences Division, Lawrence Berkeley National Laboratories, Berkeley, CA 94720, USA; ^4^Synthetic Biology Program, Space BioSciences Division, NASA AMES Research Center, Mail Stop 239-15, Moffett Field, CA 94035, USA

## Abstract

As larger-scale cloning projects become more prevalent, there is an increasing need for comparisons among high fidelity DNA polymerases used for PCR amplification. All polymerases marketed for PCR applications are tested for fidelity properties (i.e., error rate determination) by vendors, and numerous literature reports have addressed PCR enzyme fidelity. Nonetheless, it is often difficult to make direct comparisons among different enzymes due to numerous methodological and analytical differences from study to study. We have measured the error rates for 6 DNA polymerases commonly used in PCR applications, including 3 polymerases typically used for cloning applications requiring high fidelity. Error rate measurement values reported here were obtained by direct sequencing of cloned PCR products. The strategy employed here allows interrogation of error rate across a very large DNA sequence space, since 94 unique DNA targets were used as templates for PCR cloning. The six enzymes included in the study, *Taq* polymerase, AccuPrime-Taq High Fidelity, KOD Hot Start, cloned *Pfu* polymerase, Phusion Hot Start, and *Pwo* polymerase, we find the lowest error rates with *Pfu*, Phusion, and *Pwo* polymerases. Error rates are comparable for these 3 enzymes and are >10x lower than the error rate observed with *Taq* polymerase. Mutation spectra are reported, with the 3 high fidelity enzymes displaying broadly similar types of mutations. For these enzymes, transition mutations predominate, with little bias observed for type of transition.

## 1. Introduction

With the rapid pace of developments in systems biology-based research, for example, genomics, proteomics, and metabolomics, larger-scale biological discovery projects are becoming more common. Put differently, the scope of many projects has changed from the study of one/few targets to the study of hundreds, thousands, or more. An example of research that has been transformed by developments in systems biology is the cloning of expressed open reading frames (ORFs) from cDNA substrates. The traditional path for ORF cloning has usually started with experimental observations driving the identification of one or several genes of interest to a particular pathway. Cloning of target(s) then typically resulted in further refinements of pathway details and often identification of new cloning targets. With the creation and continual refinements of databases of genomic sequences, cloning now often takes place on a much larger scale. Microarray technology and DNA sequencing breakthroughs have led to a vast increase in the number of ORFs present in biological databases. Furthermore, biological observations no longer necessarily precede target identification, which now is often driven in large part by bioinformatics-based predictions and analyses. Examples of large-scale cloning efforts include structural genomics projects to systematically determine protein structures [1], pathogen ORF cloning to understand disease and therapeutic mechanisms [2], and creation of the entire human ORFeome which will further developments in basic and applied biomedical sciences [3].

DNA polymerases used to amplify targets during PCR cloning are high fidelity enzymes with error frequencies typically in the range of 10^−6^ mutations/bp amplified [4]. Minimizing PCR-generated errors is especially important for larger-scale cloning projects because, given a sufficiently large pool of target DNA sequence, even high fidelity enzymes will produce clones with mutations. There are a variety of methods to assay the fidelity of a DNA polymerase. However, error frequencies for PCR enzymes are almost always assayed using one (or a few) defined DNA target that samples a limited portion of DNA sequence space. Early studies using the relatively low-fidelity* Taq* DNA polymerase relied on the sequencing of cloned PCR products (e.g., [5, 6]). Direct sequencing of clones was a practical approach at the time due to the low fidelity of the polymerase; that is, most clones that were sequenced would contain at least one mutation.

With the introduction of higher fidelity polymerases, new screening methods were developed to rapidly interrogate large numbers of PCR products for the presence of mutations. These assays were based on a forward mutation fidelity assay developed by Kunkel and colleagues, which used a gap-filling reaction with a DNA polymerase on a* lacZ* template sequence, followed by ligation and transformation into* E. coli*. Colorimetric screening based on a functional* lacZ* gene allowed rapid identification of mutations, which were subsequently sequenced to determine the nature of the DNA alteration [7]. A similar approach was used to screen PCR products for mutations, by cloning a* lacZ* fragment amplified by PCR as opposed to simple gap filling by DNA polymerases. This method, sometimes using a different reporter gene, has been used to screen a variety of high fidelity PCR enzymes and to optimize PCR reaction conditions to minimize mutations [4, 8]. Finally, methods that rely on assaying PCR mutations based on differing chemical properties (i.e., melting temperature) of reaction products with mismatches relative to perfect duplexes have been developed and applied to a variety of enzyme systems [9, 10]. While reported fidelity values differ among research groups and assay methods, there is a general consensus that a relatively low-fidelity enzyme such as* Taq* has a fidelity value in the 10^−5^ range and higher fidelity enzymes have values that are in the 10^−6^ range (usually reported as mutations per bp per template doubling).

A tradeoff involved in using screening methods like those described above is that generally only one DNA sequence is interrogated during the assay. Additionally, limitations built into the assays further restrict the possible mutations that can be detected. For example, the assay based on screening* lacZ* gene amplification products uses a single 1.9 kb target, of which only 349 bases will produce a color change when mutated [11]. Likewise, assaying mutations based on differential duplex melting profiles is restricted to unique target sequences that are short enough, typically in the 100–300 bp range, and have thermal melting profiles that allow resolution of single mismatches [9, 10].

Because polymerase errors are known to be strongly dependent on DNA sequence context (reviewed in [12]), ideally one would use a large set of DNA sequences when measuring enzyme fidelity. This becomes especially relevant in the context of large-scale cloning projects, which involve hundreds or thousands of targets and thus contain an almost infinite DNA sequence space. To this end, we have designed and executed a study that measures enzyme fidelity by direct sequencing of cloned PCR products. Falling costs for DNA sequencing have made this method of fidelity determination practical, even for enzymes that make few mistakes. Our goals are to compare fidelity values derived from direct clone sequencing to those derived from screening-based methods, as well as to evaluate these results in the context of choosing an enzyme for a high-throughput cloning project.

## 2. Results and Discussion

To determine error rates and observe mutational spectra for a variety of DNA polymerases used in PCR cloning, we directly sequenced clones produced from 94 different plasmid templates. These plasmids, each with a unique target DNA sequence, are a subset of a larger group of glycosyltransferase clones that we have prepared from* Arabidopsis thaliana* cDNA (manuscript in preparation). The 94 plasmids have inserts with size ranging from 360 bp to 3.1 kb (median 1.4 kb) and GC content ranging from 35% to 52% (median 44%). A summary of the 6 DNA polymerases used in this study is presented in Table 1. We included* Taq* polymerase in our study because of the extensive body of literature that exists on the fidelity properties of this enzyme. The other enzymes included are all typically classified as “high fidelity” and therefore are potential candidates for large-scale cloning projects. And while comparison of fidelity values is difficult due to differences in assay and quantitation methods among different studies, a general ranking of the enzymes studied here (lowest fidelity to highest) appears to be* Taq* < AccuPrime-*Taq* < KOD ≈* Pfu *≈* Pwo *< Phusion.

**Figure 1:**
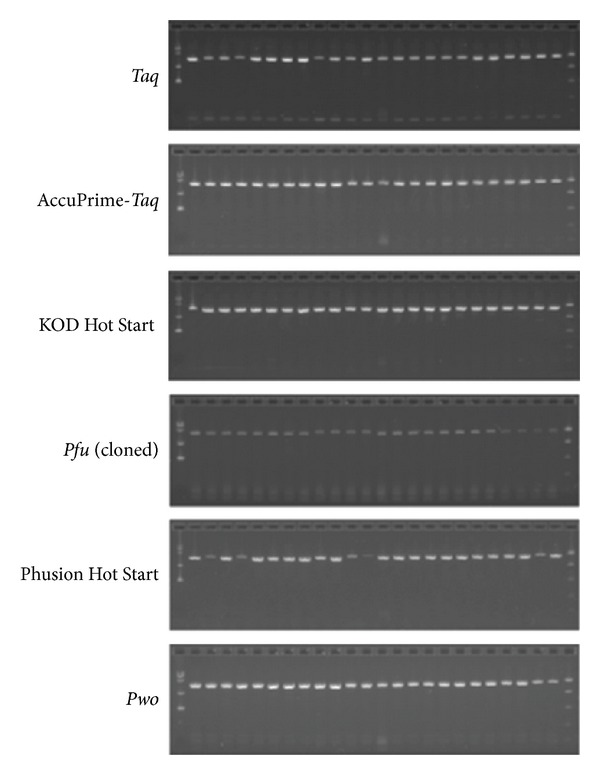
Representative agarose gel electrophoresis images of products of PCR amplification of 24 unique DNA targets, using six different enzymes. Each lane contains 1/25 of the entire PCR reaction. Expected product sizes range from 1.4 to 1.7 kb in size.

Our cloning pipeline uses recombinational insertion of purified PCR products into a plasmid vector using the Gateway cloning system, a method widely used for high-throughput cloning studies (reviewed in [17]). Since our input plasmid DNA templates were prepared using the Gateway system, the target genes of interest are all flanked by* att* recombination sequences. This allowed the use of common primers for all PCR reactions, thus eliminating the need for target-specific optimizations. Purified plasmid DNA was used as template for PCR, and in all cases vendor-recommended buffers were used. We used small amounts of plasmid template (25 pg/rxn), in order to maximize the number of doublings in the PCR reaction, and the size of insert relative to total plasmid size was taken into account to determine the amount of target fragment present in the template. The PCR protocol used 30 cycles of amplification, with an extension time of 2 minutes/cycle for targets ≤2 kb (82 of 94 targets) and 4 minutes/cycle for targets >2 kb (12 of 94 targets).  Figure 1 shows gel images for a representative set of PCR reactions for each enzyme. In all cases, a single major product band migrating at the expected size was observed. Amplification efficiency was measured by quantitation of PCR product using a dsDNA-specific dye and calculating the fold-amplification based on a known quantity of input DNA template. The fold-amplification is used to determine the number of template doublings that occurred during PCR. As reported in Table 2, amplification efficiency values were fairly uniform for all samples within a plate. We observe similar amplification efficiencies between different enzymes, with the exception that we routinely observed fewer template doublings in reactions with* Pfu* polymerase. We have kept thermocycling protocols constant for all enzymes, and thus it is possible that some parameters were not optimal for amplification by* Pfu*.

Following amplification, PCR products were purified by precipitation with PEG/MgCl_2_, which is known to selectively fractionate DNA on the basis of size [18], to remove short products <300 bp in size. This precipitation step can be performed in 96-well plate format, which is a requirement when the number of samples becomes large. We have adopted this protocol for routine use and have observed a higher efficiency for insertion of correct-size DNA into the vector compared to purification using kit-based PCR purifications, which typically have size cutoffs of ~100 bp (data not shown). In the case where off-target PCR products of >300 bp are present, gel extraction is used to isolate the desired product. Purified PCR products were incubated with vector DNA and BP Clonase II and transformed into competent cells. Three colonies per plate were picked and grown up in 96-well plates, and cultures were screened for correct-size insert by colony PCR. Insertion efficiency values for BP Clonase II, expressed as the average number of clones having an insert at/near the expected size (out of 3 colonies screened per transformation), were typically 80–90% (data not shown). For each target, one or more clones for each target containing a correct-size insert (if obtained) were cultured and used for DNA sequencing.

For method validation purposes, we used* Taq* DNA polymerase, a Family A DNA polymerase and the enzyme used in the earliest PCR experiments [6]. As an early workhorse in PCR technology,* Taq* polymerase has been studied extensively for purposes of fidelity determination.* Taq* DNA polymerase lacks a 3′→5′ exonuclease activity and thus is unable to correct misincorporated nucleotides that occur during DNA synthesis. Various assays have been used to assay* Taq* fidelity, and, depending on the method used, error rate values (expressed as mutations per base pair per template duplication) for* Taq* polymerase range from ~1 × 10^−5^ (e.g., [4, 19]) to 2 × 10^−4^ (e.g., [7, 20]). Furthermore, the mutational spectrum of* Taq* polymerase has been characterized, with A•T → G•C transitions predominating due to the propensity for the enzyme to misincorporate incoming dCTP with a template thymine nucleotide [6, 9, 21].

By direct sequencing of clones from two independent PCR experiments with* Taq* polymerase, we observed 99 unique mutations out of >100 kbp of target DNA sequence. The type and number of individual mutations are listed in Table 3. Given the amplification efficiency of each PCR reaction, the error rate (average of 2 experiments) for* Taq* polymerase is 4.3 × 10^−5^ ± 1.8 mutations/bp per template duplication. This value is in excellent agreement with other published values for this enzyme, and the relatively high variance suggests that calculated error values differing by up to 2-fold are probably not significant relative to the experimental noise. The majority of the mutations (67 of 99) are A•T → G•C transitions, which could result from either incoming dCTP mispairing with template A or incoming dGTP mispairing with template T. Transitions of the G•C→A•T type, resulting from either incoming TTP mispairing with template G or incoming dATP mispairing with template C, are the second most prevalent mutation (28 of 99). There were 3 transversion mutations, with 1 A•T→T•A and 2 A•T→C•G changes. Overall, the spectrum of the base substitution mutations agrees well with previous observations on* Taq* polymerase reported in the literature [7]. There was only one insertion or deletion (indel) mutation observed in our data set, a single T deletion in a T_3_ template sequence.* Taq* polymerase has been reported to produce indel mutations with a significant frequency, as much as approximately 25% of total mutations, with all occurring in homopolymeric runs [7]. Since our target pool contains 1481 instances of homopolymer runs of at least 4 bp, we suspect that other differences between the earlier assay conditions and those used here explain the discrepancy. Specifically, the earlier experiments were performed with elevated magnesium (10 mM versus 1.5 mM used here) and elevated dNTP levels (1 mM versus 0.2 mM used here). Both elevated magnesium and dNTP levels were subsequently shown to elevate frameshift (indel) mutations preferentially relative to base substitution mutations [21].

An important control for these experiments is necessitated by the method used to generate template for DNA sequencing. For larger-scale cloning projects, DNA sequencing using cell culture is advantageous because of the saving in time and resources relative to purifying plasmid DNA. However, sequencing using cell culture requires a PCR or another amplification step, and this step could in principle be a source of “additional mutation.” To address this directly, we sequenced miniprep DNA prepared from a subset of clones produced with* Taq* polymerase. Every one of the fourteen mutations detected in the subset using cell culture as the source for sequencing template was also observed when sequencing from plasmid DNA template (data not shown). We conclude that our method has a false positive rate of <7% (1/14) and is acceptable for assaying PCR-induced mutations. Furthermore, based on our results with* Taq* polymerase, we conclude that our method for fidelity determination gives results in excellent agreement with other studies and is thus an accurate measure of polymerase accuracy.

Our results indicate that 3 of the enzymes included in the study,* Pfu* polymerase, Phusion Hot Start, and* Pwo* polymerase, have error rates that are significantly lower than the others. This is consistent with previous findings demonstrating very high fidelity PCR amplification for these enzymes. Interestingly, error frequency values for these three enzymes are extremely similar to each other, approximately 2-3 × 10^−6^ mutations/bp/template doubling. The slight error frequency value differences are probably not significant, given that the small number of mutations is produced by these high fidelity polymerases in addition to the experimental variability discussed above for the results with* Taq*. Given the costs of cloning and sequencing and finite research budgets, mutation detection by DNA sequencing of clones generates a relatively small data set of mutations when the enzyme fidelity is high. This is a drawback to our assay, and despite the fact that DNA sequencing costs continue to drop screening bacteria is still a far more economical method of interrogating a large number of clones. For all mutant clones produced by* Pfu*, Phusion Hot Start, and* Pwo* polymerases, samples were resequenced to rule out sample processing or DNA sequencing as a source of error. In all cases, the original mutation was present, confirming the PCR reaction as the most likely source of the mutation. From the standpoint of use in a large-scale cloning project, any one of these enzymes would be acceptable, judged on the criteria of minimizing error rate. Other factors need to be considered of course, such as amplification efficiency, mutation spectra, performance with high GC content templates, and cost, to name a few. As far as mutation spectra, the 3 high fidelity polymerases all produced predominantly (>75%) transition mutations, with no significant template bias. With Phusion enzyme, we observed 15% (2/13) indel mutations, which are problematic for cloning applications where translation reading frame should be maintained. Both indel mutations occurred in repeat regions, with one being an A insertion into an A_3_ template sequence and the other being a (TCT) deletion within a (TCT)_5_ template sequence. This result was unexpected in light of the high processivity of Phusion polymerase relative to other commonly used PCR enzymes (vendor website). Because multiple studies have found that increased polymerase processivity reduces the frequency of slippage mutations that result in indel mutations [22, 23], we expected Phusion to produce the fewest of this class of errors. It should be noted, however, that this conclusion is based on a small sample size and a larger number of mutations should be analyzed for confirmation.

It was interesting to us that none of the enzymes tested here was found to have an error rate below ~2 × 10^−6^. Other studies in the literature have reported sub-10^−6^ error frequencies for PCR enzymes, 6.5 × 10^−7^ [10] for* Pfu* polymerase assayed by differential duplex T_m_ measurement and 4.2 × 10^−7^ for Phusion, using HF buffer assayed with a method called BEAMING [16]. For the study of Phusion fidelity, the PCR used a different buffer than the one employed here, which according to the vendor does result in a 2-3-fold lower error rate. In addition, that study uses the BEAMING method, an extremely sensitive flow cytometric protocol that screens large numbers of beads that contain PCR products for the presence of nucleotide variations. However, only one specific mutation, a G•C → A•T mutation at a single position, was interrogated in that study. Thus, while the assay is extremely sensitive for detection of defined mutations, results obtained with the BEAMING method for mutation frequency at a single position may not necessarily reflect the fidelity properties of an enzyme for much larger sequence spaces. For the study on* Pfu* error rate, several fundamental methodological differences are present: in the earlier study, the PCR was performed under “almost anaerobic” conditions with significantly shorter cycling times, the target size was limited to 93 bp, and mutation detection relied on a physiochemical method: separation and isolation of PCR products containing mismatches by capillary electrophoresis [10]. And while this method has been successfully used in the detection of rare mutations in mitochondrial DNA samples from normal and cancer tissues [24], the requirement for a mutation to result in a molecule with an altered melting profile may bias the number of mutations that can be detected. A major discrepancy between our results and those from this earlier report on* Pfu* fidelity, which may be connected to the differing mutation detection methodologies, can be seen in the mutation spectra results in Table 3. We observed ~90% (8 of 9) transition mutations, with a slight bias for G•C → A•T alterations. In contrast, the study using capillary electrophoresis for detection resulted in predominantly (3/5) transversion mutations, with a single A•T → G•C transition and a single 1 bp deletion mutation. Transversion mutations require the polymerase to synthesize either a purine•purine or a pyrimidine•pyrimidine mismatch, both of which are significantly disfavored relative to the different purine•pyrimidine mismatches in Family B polymerases, including* Pfu* polymerase [25, 26]. Because the types of mutations we observe are consistent with previously reported mutational spectra for other Family B polymerases, we believe our method has detected polymerase errors in a bias-free fashion.

The other two enzymes included in our study, KOD polymerase and AccuPrime-*Taq* High Fidelity, have fidelity values intermediate between* Taq* polymerase and the higher fidelity enzymes. The error rate observed for KOD polymerase was only ~4-fold lower than that of* Taq* polymerase and ~2.5-fold higher than for* Pfu* polymerase. The initial report on fidelity of KOD polymerase, a Family B/pola-like polymerase from* Thermococcus kodakaraensis* KOD1, reported an error rate very slightly lower than* Pfu* polymerase and ~4-fold lower than for* Taq* polymerase [13]. That study used a forward mutation assay (not PCR), expressed fidelity simply as the ratio of white colonies to blue with no accounting for PCR amplification efficiency, and used experimental conditions (Mg^2+^ concentration) that differ significantly from typical PCR conditions. A subsequent study measuring fidelity under PCR conditions, using a different reporter gene but still a simple ratio of mutant to wild-type colonies, reported error rates ~50x lower than those with* Taq* and marginally lower than those for* Pfu* polymerase [14]. In neither of those studies was there a report of the molecular changes leading to mutant colonies. The large difference between these two results, which are from the same research group, serves to highlight the difficulties in making comparisons between studies where there are significant methodological differences. In the present study, we find that the mutation spectrum for KOD polymerase is similar to the other B-family polymerases (*Pfu, Pwo,* and Phusion) assayed here. As shown in Table 3, transitions predominate (14 of 16 mutations), with a slight bias (64%) for A•T → G•C mutations.

For the PCR performed with AccuPrime-*Taq* High Fidelity system, we observed a 3-fold improvement in fidelity relative to* Taq* polymerase. According to the vendor, AccuPrime-*Taq* High Fidelity is an enzyme blend that contains* Taq* polymerase, a processivity-enhancing protein, and a higher fidelity proofreading polymerase from* Pyrococcus* species* GB-D*. The lower error rate seen with AccuPrime-*Taq* most likely arises from the* GB-D* polymerase editing mistakes introduced by* Taq* polymerase as opposed to enhanced processivity since increased processivity has been shown to have no significant effect on base substitution errors [22, 27]. The mutation spectrum of the blend is almost identical to that seen with* Taq* polymerase alone, with transitions predominant and a significant bias for A•T → G•C changes (71% for AccuPrime-*Taq* versus 73% for* Taq*). However, it should be noted that a study on the mutation spectra of* GB-D* DNA polymerase (commercially available as Deep Vent) found A•T → G•C transitions to be the predominant mutation [28]. Detailed analysis on the contribution of each enzyme to the overall mutation spectrum is also precluded by the proprietary enzyme formulation used by the vendor.

In summary, we have used direct DNA sequencing of cloned PCR products to assay polymerase fidelity and evaluate other aspects of enzyme suitability for large-scale cloning projects. Based on minimizing PCR errors,* Pfu* polymerase,* Pwo* polymerase, and Phusion all produce acceptably low levels of mutations. Phusion was observed to produce more indel mutations than* Pfu* or* Pwo* polymerases, although the total number of mutations was limited. This type of mutation is particularly problematic for ORF cloning projects and should be taken into account in the process of enzyme selection. Aside from fidelity considerations, amplification efficiency values were significantly higher for Phusion and* Pwo* compared to* Pfu*, although further optimization of the PCR reaction for* Pfu* would likely improve efficiency values. Likewise, for cloning projects where targets are either very long or very highly GC-rich fidelity may be of lesser importance relative to the ability to amplify “difficult” target DNA. And finally, since the application space for PCR technology is huge, with cloning representing only a small fraction, enzymes other than those studied here need to be compared and evaluated based on project-specific needs and challenges.

## 3. Materials and Methods

### 3.1. PCR Reactions

All enzymes and reaction buffers were from commercial sources: Fermentas (*Taq* polymerase), Invitrogen/Life Technologies (AccuPrime-*Taq*), EMD Chemicals/Novagen (KOD Hot Start), Agilent (cloned* Pfu* polymerase), Finnzymes (Phusion Hot Start), and Roche (*Pwo* polymerase). PCR reactions were carried out in a final volume of 50 *μ*L using buffer conditions and enzyme amounts recommended by the vendor. For reactions with Phusion, the GC buffer was used. In all cases, reactions included 0.2 mM each dNTP (Fermentas) and 0.2 mM each primer (IDT) with the sequences (5′ to 3′) GGGGACAAGTTTGTACAAAAAAGCAGGCTTCACC for the forward primer and GGGGACCACTTTGTACAAGAAAGCTGGGTC for the reverse primer. Template for PCR reactions was miniprep plasmid DNA, with each plasmid template containing a unique target sequence of known sequence and size, ranging from 0.3 to 3 kb. The target insert was cloned in between the* att* sites of a pDONR vector, allowing the use of a common primer set for all plasmids. Each PCR reaction contained 0.025 ng plasmid DNA, quantitated using the PicoGreen DNA quantitation reagent (Invitrogen/Life Technologies), and thus the amount of input target (*i*) was calculated as *i* = 0.025 ng × (size of target ÷ (size of target + size of plasmid)). The thermocycling protocol for all reactions with target length ≤2 kb was 5 minutes, 95°C, then 30 cycles of 15 seconds, 95°C → 30 seconds, 55°C → 2 minutes, 72°C, and finally 7 minutes at 72°C. For the targets >2 kb in size, the 2-minute extension step was extended to 4 minutes. For analysis of PCR products by gel, 2 *μ*L of each PCR reaction was run on a 2% agarose eGel (Invitrogen/Life Technologies) run according to vendor recommendations.

### 3.2. Quantitation of PCR Reactions

Efficiency of PCR amplification was determined by measuring the amount of product using a modified PicoGreen dsDNA quantitation assay. This method was facilitated by optimizing the PCR reaction to produce a single product band (Figure 1). Using a Biomek FX-P (Beckman) automated liquid handing system, 5 *μ*L of each PCR reaction was diluted 50-fold in TE buffer (pH 8) into a new 96-well plate. From this plate, 5 *μ*L from each well was mixed with 195 *μ*L of PicoGreen solution, a 500-fold dilution of dye in TE (pH 8). Fluorescence measurements were taken with a Paradigm (Beckman) plate reader. Background fluorescence was determined from a PCR reaction that contained no template DNA. Following background subtraction, DNA concentration was determined by comparing fluorescence readings to those obtained with a standard curve using DNA of known concentration supplied with the dye. Extent of target amplification (*e*) is calculated as* e* = (ng DNA after PCR) ÷ (ng of target DNA input), and the number of template doublings during PCR (*d*) can be calculated as *e* = 2^*d*^.

### 3.3. Cloning of PCR Products

PCR reactions were purified in 96-well plate format by the addition of PEG 8000 and MgCl_2_ to final concentrations of 10% and 10 mM, respectively, directly to each well of the PCR plate using a multichannel pipettor. The plate was spun at 4000 rpm for 60 minutes at room temperature, and the supernatant was discarded. Pellets were washed two times with cold isopropanol, air-dried, and resuspended in 25 *μ*L TE (pH 8). This protocol resulted in excellent yields (50–75%) of PCR products, with no products <300 bp, as judged by gel electrophoresis. Purified PCR products were cloned into a pDONR223 vector (a generous gift of Drs. Dominic Esposito and Jim Hartley, NCI, Frederick, MD) using BP Clonase II (Invitrogen/Life Technologies). Clonase reactions were assembled using a multichannel pipettor in 96-well PCR plates in a 5 *μ*L volume and contained 75 ng pDONR223, 1 *μ*L purified PCR product (typically 50–150 ng DNA), and 1 *μ*L BP Clonase II. Sealed plates were incubated at least 16 hours at 25°C, and 1 *μ*L of each reaction was immediately (no proteinase K treatment) used to transform either 25 *μ*L or 50 *μ*L of competent TOP10 cells (Invitrogen/Life Technologies). Following heat shock and recovery, following addition of 250 *μ*L of SOC media, 100 *μ*L of cells was plated on LB plates containing 50 mg/mL spectinomycin. Equivalent numbers of colonies were observed in transformations using 25 *μ*L or 50 *μ*L of frozen competent cells, and control BP reactions lacking BP Clonase II or PCR product resulted in no transformants.

### 3.4. Screening of Transformants

Three colonies from each transformation plate were picked and cultured in 96-well plates (Costar 3788) sealed with gas-permeable membrane, with each colony incubated in 150 mL of LB media with 50 mg/mL spectinomycin and 10% glycerol. After overnight incubation at 37°C (no shaking), 1 *μ*L of each culture was used to screen by colony PCR for the presence of insert with expected size. Colony PCR reactions (25 mL) used the same primers used for cloning at a final concentration of 0.1 mM each, with 30 amplification cycles as described above, with* GoTaq* polymerase (Promega). Reactions were analyzed by agarose gel electrophoresis, and the presence of a band at or near the expected size was scored as a “hit.” The number of hits (0–3) for each target was determined, and an average number of hits per target for each plate were determined and used as a measure of Clonase reaction efficiency.

### 3.5. Clone Sequencing

In cases where Clonase efficiency values were >66%, average of at least 2 hits out of 3 colonies screened, the entire liquid culture plate was replicated with a 96-pin replicator onto an agar plate with the same dimensions as a 96-well plate. The plate was immediately submitted to an outside vendor (Quintarabio, Berkeley, CA), and after growth overnight sequencing was performed on amplified DNA from each clone. If Clonase efficiency values were <66% (*Taq* and* Pfu* polymerase reactions), a rearray step was added, using a Qpix2 colony picking robot (Genetix) to maximize the number of clones with correct-size insert on one plate. For comparing sequencing results using cells versus miniprep DNA, one plate of colonies picked from a* Taq* cloning reaction was replicated into a 96-well deep well plate with 800 mL media per well and grown overnight with shaking at 300 rpm. Cells were pelleted, and DNA was prepared using a Qiaprep 96 Turbo Miniprep Kit (Qiagen). Eluted DNA was submitted directly for sequencing.

## Figures and Tables

**Table 1:** Published fidelity (error rate) values for DNA polymerases used in this study. Due to the numerous methodological and analytical differences among studies, values are often reported as ranges. Furthermore, the references listed are meant to provide representative, but not necessarily exhaustive, documentation for error rate values. All values are given using *Taq* as the reference (1x).

Enzyme	Published error rate (errors/bp/duplication)	Fidelity relative to *Taq *	References
*Taq *	1–20 × 10^−5^	1x	[4, 7, 9]
AccuPrime-*Taq*, HF	N/A	9x better	Vendor website
KOD	N/A	4x better, 50x better	[13, 14]
*Pfu *	1-2 × 10^−6^	6–10x better	[4, 15]
Phusion Hot Start	4 × 10^−7^ (HF buffer), 9.5 × 10^−7^ (GC buffer)	>50x better (HF buffer), 24x better (GC buffer)	[16], Vendor website

**Table 2:** Error rate values for six PCR enzymes included in this study are presented. Each enzyme was used in two independent PCR reactions. Average doublings/PCR reaction (*d*) is the average of doubling values for each of the 94 PCR reactions in one plate, where doublings are calculated from the formula 2^*d*^ = (ng DNA after PCR/ng DNA input). Error rate (*f*) is calculated as *f* = *n*/*S* (target size ×  *d*), where *n* is the number of mutations observed for all clones that were sequenced and the (target size ×  *d*) for each target that was cloned.

Enzyme	Expt.	Avg. doublings/PCR reaction	Number of clones sequenced	Total bp sequenced	Number of mutations observed	Error rate
*Taq *	1	20.5 ± 1.2	65	8.8 × 10^4^	54	3.0 × 10^−5^
	2	16.7 ± 0.7	37	4.7 × 10^4^	45	5.6 × 10^−5^

AccuPrime-*Taq *	1	17.0 ± 1.2	75	1.0 × 10^5^	18	1.0 × 10^−5^
	2	16.9 ± 0.6	N.D.	N.D.	N.D.	N.D.

KOD	1	20.8 ± 1.5	70	1.0 × 10^5^	16	7.6 × 10^−6^
	2	17.6 ± 0.8	N.D.	N.D.	N.D.	N.D.

*Pfu* (cloned)	1	16.5 ± 1.1	151	2.0 × 10^5^	9	2.8 × 10^−6^
	2	12.0 ± 1.8	N.D.	N.D.	N.D.	N.D.

Phusion	1	21.0 ± 1.9	175	2.4 × 10^5^	13	2.6 × 10^−6^
	2	16.6 ± 1.1	N.D.	N.D.	N.D.	N.D.

*Pwo *	1	22.5 ± 1.2	170	2.4 × 10^5^	13	2.4 × 10^−6^
	2	17.6 ± 0.6	N.D.	N.D.	N.D.	N.D.

N.D.: not determined.

**Table 3:** Mutational spectra of six different PCR enzymes are presented. Results with *Taq* polymerase are mutations observed in sequencing clones from two independent PCR reactions. For indel mutations, the type of insertion or deletion is indicated.

	*Taq *	AccuPrime-*Taq*, HF	KOD	*Pfu *	Phusion	*Pwo *
Transitions						
A•T→G•C	67	12	9	3	6	6
G•C→A•T	28	5	5	5	5	4
Transversion						
A•T→T•A	1	1	1	1		
A•T→C•G	2					
G•C→T•A						3
Indels	1 (T del.)		1 (T del.)		2 (A ins., TCT del in (TCT)_5_ run)	

Insertion = ins.; deletion = del.
